# The NS1 protein of the parvovirus MVM Aids in the localization of the viral genome to cellular sites of DNA damage

**DOI:** 10.1371/journal.ppat.1009002

**Published:** 2020-10-16

**Authors:** Kinjal Majumder, Maria Boftsi, Fawn B. Whittle, Juexin Wang, Matthew S. Fuller, Trupti Joshi, David J. Pintel

**Affiliations:** 1 Department of Molecular Microbiology and Immunology, University of Missouri School of Medicine, Columbia, Missouri, United States of America; 2 Christopher S. Bond Life Sciences Center, University of Missouri, Columbia, Missouri, United States of America; 3 Pathobiology Area Graduate Program, University of Missouri, Columbia, Missouri, United States of America; 4 Department of Electrical Engineering and Computer Science, University of Missouri, Columbia, Missouri, United States of America; 5 Ultragenyx Gene Therapy, Cambridge, Massachusetts, United States of America; 6 Department of Health Management and Informatics, University of Missouri School of Medicine, Columbia, Missouri, United States of America; 7 MU Institute of Data Science and Informatics, University of Missouri, Columbia, Missouri, United States of America; Stony Brook University, UNITED STATES

## Abstract

The autonomous parvovirus Minute Virus of Mice (MVM) localizes to cellular DNA damage sites to establish and sustain viral replication centers, which can be visualized by focal deposition of the essential MVM non-structural phosphoprotein NS1. How such foci are established remains unknown. Here, we show that NS1 localized to cellular sites of DNA damage independently of its ability to covalently bind the 5’ end of the viral genome, or its consensus DNA binding sequence. Many of these sites were identical to those occupied by virus during infection. However, localization of the MVM genome to DNA damage sites occurred only when wild-type NS1, but not its DNA-binding mutant was expressed. Additionally, wild-type NS1, but not its DNA binding mutant, could localize a heterologous DNA molecule containing the NS1 binding sequence to DNA damage sites. These findings suggest that NS1 may function as a bridging molecule, helping the MVM genome localize to cellular DNA damage sites to facilitate ongoing virus replication.

## Author summary

Parvoviruses are among the simplest of viruses, depending almost exclusively on host cell factors to successfully replicate. We have previously shown that the parvovirus Minute Virus of Mice (MVM) establishes replication centers at sites that are associated with cellular regions of DNA damage. These sites are primed to contain factors necessary to efficiently initiate vigorous virus lytic infection. The process by which viral proteins and viral DNA specifically localize to these sites has previously remained unknown. In this study we show that the essential viral protein NS1 possesses the intrinsic ability to localize to cellular sites of DNA damage. Additionally, wild-type NS1, but not its DNA binding mutant, could localize to sites of DNA damage both the MVM genome, or a heterologous DNA molecule engineered to contain NS1 binding sites. This work provides the first evidence that NS1 may function as a bridging molecule to localize the MVM genome to cellular sites of DNA damage to facilitate ongoing replication.

## Introduction

Parvoviruses are non-enveloped icosahedral viruses that contain a linear genome of single-stranded DNA [1]. The prototype parvovirus Minute Virus of Mice (MVM) is lytic in murine cells and transformed cells of multiple species including human, and replicates autonomously during S phase through rolling hairpin replication [2,3]. The MVM genome is approximately 5 kb long with inverted terminal repeats at either end which serve as replication origins. The virus encodes two non-structural phosphoproteins, NS1 and NS2. NS1 is essential for numerous facets of viral replication in all permissive cell types, whereas NS2’s function during infection is less clearly defined, and is essential only in murine cells [4,5].

Following S phase entry, the single-stranded MVM genome is amplified by alternating monomeric and concatemeric duplex replicative intermediates, utilizing cellular DNA polymerase δ. The unit-sized double stranded intermediate also serves as the primary template for the expression of viral genes [6,7]. NS1 molecules can dimerize asymmetrically on the octameric consensus motif ACCAACCA present at 14 locations on the viral genome [8–10]. Besides binding to its consensus DNA motif, NS1 facilitates genome replication as a site- and strand-specific nickase and helicase, and covalently binds to the 5’ end of the viral genome during replication [6,11–14].

MVM establishes replication factories in the nucleus (termed Autonomous Parvovirus Associated Replication, or APAR bodies), where ongoing genome replication and expression take place [15–18]. In addition to viral DNA, NS1 colocalizes there with replication factors such as PCNA, RPA and DNA polymerases α and δ in viral replication centers [15,16,19]. At early stages of infection, MVM induces cellular DNA damage which results in a DDR in host cells, driven by signaling from the ATM kinase and virally mediated inactivation of the CHK1 kinase [15,19]. APAR bodies are also sites where cellular DNA Damage Response (DDR) sensor and response proteins accumulate and associate with replicating viral DNA [15,20,21]. As virus replication proceeds, MVM programs the degradation of p21 through the CRL4^Cdt2^ ubiquitin ligase pathway, and downregulates Cyclin B1 expression in part by dysregulating the transcription factor FOXM1, ultimately resulting in a potent pre-mitotic cell cycle block [22–25].

To establish successful infection, MVM must co-opt factors essential for virus expression and replication while simultaneously evading the sentinels of genome integrity. Importantly, rather than merely recruiting essential proteins to replication centers, the MVM genome localizes to cellular genomic sites that contain high concentrations of replication and repair proteins. High-throughput chromosome conformation capture assays (which we have termed V3C-seq) have mapped the primary localization sites of the replicating MVM genome with cellular sites of DNA damage replete with such factors, many of which coincide with Early Replicating Fragile sites (ERFs, [21,26]). As replication proceeds, MVM induces and then spreads to additional sites of DNA damage. However, the molecular machinery that enable the MVM genome to localize to cellular DDR sites, and potentially aid in its expansion, remains unknown. A potential factor that could enable proper viral genome localization is NS1, which is both bound to, and essential, for the replicating MVM genome.

Here, we show that NS1 expressed in the absence of infection can localize to cellular sites of DNA damage that have been induced with either focused laser micro-irradiation, or chemically induced with hydroxyurea or doxorubicin. Mutant NS1, which is incapable of dimerizing and subsequently binding to its cognate ACCAACCA sequence also localizes to cellular DDR sites. Many of these sites were identical to those occupied by the virus during infection. However, localization of the MVM genome to sites of DNA damage could occur only when wild-type NS1, but not its DNA-binding mutant was expressed. Additionally, wild-type NS1 protein, but not its DNA binding mutant, could localize a heterologous DNA molecule containing the NS1 binding sequence to sites of DNA damage. These findings suggested that NS1 may function as a bridging molecule which helps localize the MVM genome to cellular sites of DNA damage to facilitate ongoing virus replication.

## Results

### MVM NS1 localizes to induced cellular sites of DNA damage during infection and overexpression

**Fig 1:**
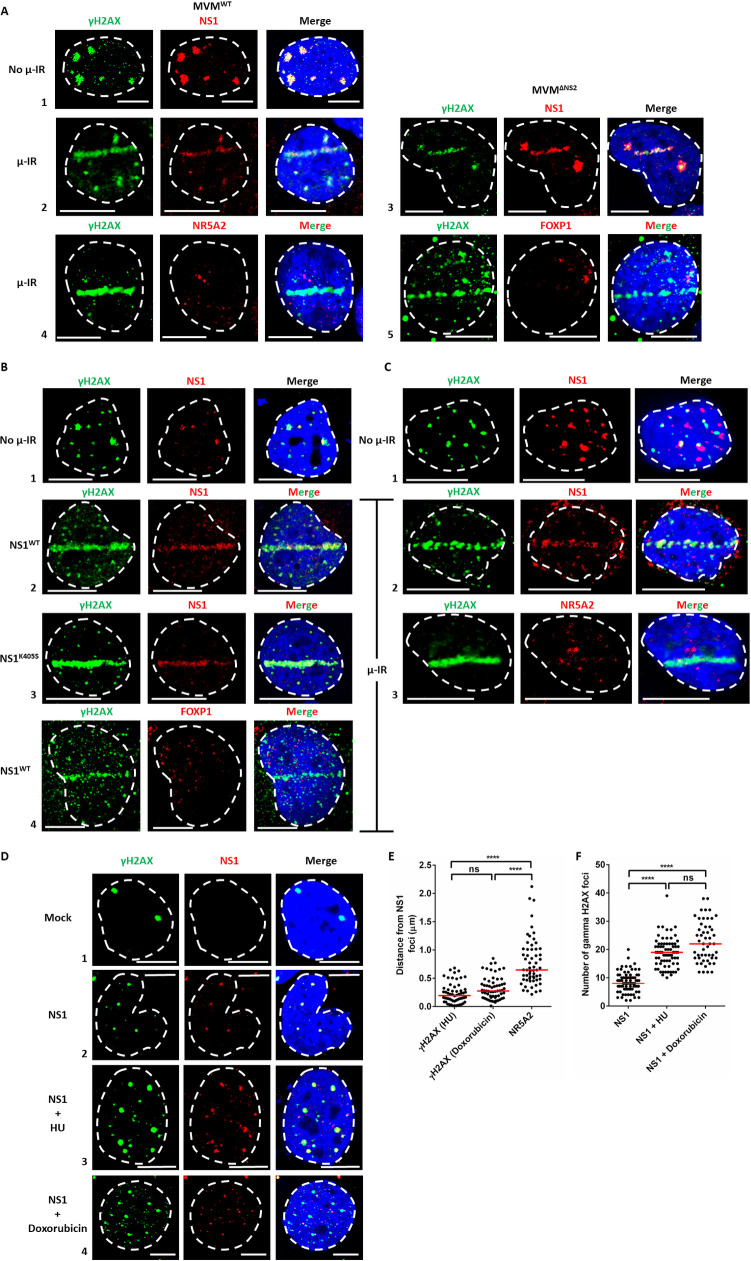
MVM NS1 localizes to induced cellular sites of DNA damage during infection and overexpression. (A) Laser micro-irradiation followed by immunofluorescence analysis of U2OS osteosarcoma cells was performed during infection with MVMp (MVM^WT^; panels 1,2,4,5) and MVMp1989 variant (MVM^ΔNS2^; panel 3, [66]) of MVM for 16 hours. The induced DDR site was monitored by γH2AX staining (green) and the viral non-structural protein was visualized by NS1 staining (red). The host transcription factors FOXP1 and NR5A2, which do not have strong binding sites on the MVM genome, were monitored as controls (red). The cell nuclei were visualized by DAPI staining and the nuclear periphery was demarcated by dashed white line. White bars in figure inset represent 10 microns. Data is representative of 3 independent experiments, each imaging at least five fields of view containing 4–5 nuclei. (B) U2OS cells were transfected with the indicated NS1 expressing vector with LipoD293 for 16 hours, DDR induced by laser micro-irradiation and processed for immunofluorescence by co-staining for γH2AX (green) and NS1 protein (red). The cell nuclei were visualized by DAPI staining and the nuclear periphery was demarcated by dashed white line. White bars in figure inset represent 10 microns. Data is representative of 3 independent experiments, each imaging at least five fields of view containing 4–5 nuclei. (C) Murine A9 fibroblasts which stably express NS1^WT^ were induced with doxycycline for 24 hours before focused DDR induction by laser micro-irradiation. Samples were then co-stained for γH2AX (green) and the indicated proteins (red). The cell nuclei were visualized by DAPI staining and the nuclear periphery was demarcated by dashed white line. White bars in figure inset represent 7.5 microns. Data is representative of 3 independent experiments, each imaging at least five fields of view containing 4–5 nuclei. (D) U2OS cells were transfected with NS1 expressing vector for 12 hours and induced with 1 mM Hydroxyurea or 200 nM Doxorubicin for 4 hours to chemically induce DNA damage. Samples were collected by CSK pre-extraction at 16 hours post-expression and processed for immunofluorescence by co-staining for DNA damage sites, visualized by γH2AX (green) and NS1 (red). The cell nuclei were visualized by DAPI staining and the nuclear periphery was demarcated by dashed white line. White bars in figure inset indicate 10 microns. (E) The distance between the center of mass of the fluorescence of γH2AX foci and NS1 foci were quantified for multiple nuclei imaged across 3 independent experiments using Leica LAS X image processing software. (F) The number of γH2AX foci were quantified in NS1 expressing cells over multiple fields of view in 3 independent experiments. Statistical analysis was performed using One-way ANOVA, multiple comparisons test, with statistical significance designated by ****, which represents p <0.0001. ns denotes not statistically significant.

As shown in Fig 1A, during wild-type MVM (MVM^WT^) infection in U2OS osteosarcoma cells, which are fully permissive for MVM in an NS2-independent manner, NS1 co-localized with cellular sites of DNA damage monitored by γH2AX staining, as a part of APAR bodies (Fig 1A, panel 1), consistent with previously published observations [15,20,21]. Upon induction of DNA damage by laser micro-irradiation in U2OS cells 16 hours post infection (16 hpi), a significant portion of NS1 relocalized to the laser micro-irradiated stripe as monitored by γH2AX staining (Fig 1A, panel 2). When U2OS cells were infected with an MVM mutant unable to generate NS2 (MVM^ΔNS2^), NS1 also colocalized with micro-irradiated DDR sites (Fig 1A, panel 3), suggesting that NS2 was not required for this localization. The unrelated transcription factors NR5A2 and FOXP1, which do not have strong binding sites on the MVM genome, did not relocalize to the laser micro-irradiated sites, and instead remained distinct from the γH2AX staining pattern (Fig 1A, panels 4 and 5 respectively).

Following induction of DNA damage by laser micro-irradiation, wild-type NS1, expressed individually from a CMV-driven expression vector also relocalized to γH2AX stripes from APAR-like foci (Fig 1B, compare panels 1 and 2), suggesting that NS1 could localize to laser micro-irradiated sites independently of the replicating virus and even though the expressed NS1 gene contains NS1 binding sites. NS1 possesses a dimerization-dependent DNA binding and helicase activity which requires binding and hydrolysis of ATP [8]. Mutation of NS1 lysine 405 (NS1^K405S^) interferes with its ability to dimerize and prevents binding to its cognate ACCAACCA site [8,27]. Surprisingly, NS1^K405S^, overexpressed transiently in U2OS cells, still efficiently relocalized to laser micro-irradiated sites, while non-MVM binding control FOXP1 remained distinct from γH2AX in these cells (Fig 1B, panels 3 and 4).

To further analyze NS1’s relocalization to laser stripes, we generated stable A9 cell lines which inducibly express just NS1 [28]. Focused DNA damage generated by laser micro-irradiation 24 hours post-induction of NS1 with doxycycline also led to NS1’s relocalization to micro-irradiation induced DNA breaks (Fig 1C, compare panels 1 and 2), whereas control NR5A2 remained distinct from the induced damage sites in these cells (Fig 1C, panel 3).

We next monitored NS1’s localization to punctate sites of DNA damage induced either by pulsing cells with hydroxyurea (HU), or doxorubicin, which generate DNA damage *via* replication stress, by inhibiting ribonucleotide reductase (HU), or hampering topoisomerase II progression [29,30]. U2OS cells expressing CMV-driven NS1 formed foci which colocalized with γH2AX in the absence of treatment (Fig 1D, panels 1 and 2); pulsing these cells with HU or doxorubicin led to the formation of more, and larger γH2AX foci, to which NS1 staining colocalized (Fig 1D, panels 3 and 4). Quantification of multiple fields of view demonstrated that the distance between the center of mass of the NS1 and γH2AX foci in the presence of DNA damaging agents was significantly smaller (closer) than their distance from control NR5A2 foci (Fig 1E). Additionally, induction of replication stress led to a greater number of γH2AX foci per nucleus (Fig 1F), indicating that the cellular target regions where NS1 could potentially relocalize increased in the presence of generalized DNA damage.

### Analysis of NS1 localization and relocalization to DDR sites at the genomic level

To characterize how individually expressed NS1 localizes and can be re-localized to cellular DNA damage sites throughout the genome, and in higher resolution, we performed ChIP-seq for NS1 and γH2AX in stable A9 fibroblasts inducibly expressing NS1 with doxycycline, in the presence and absence of HU.

**Fig 2:**
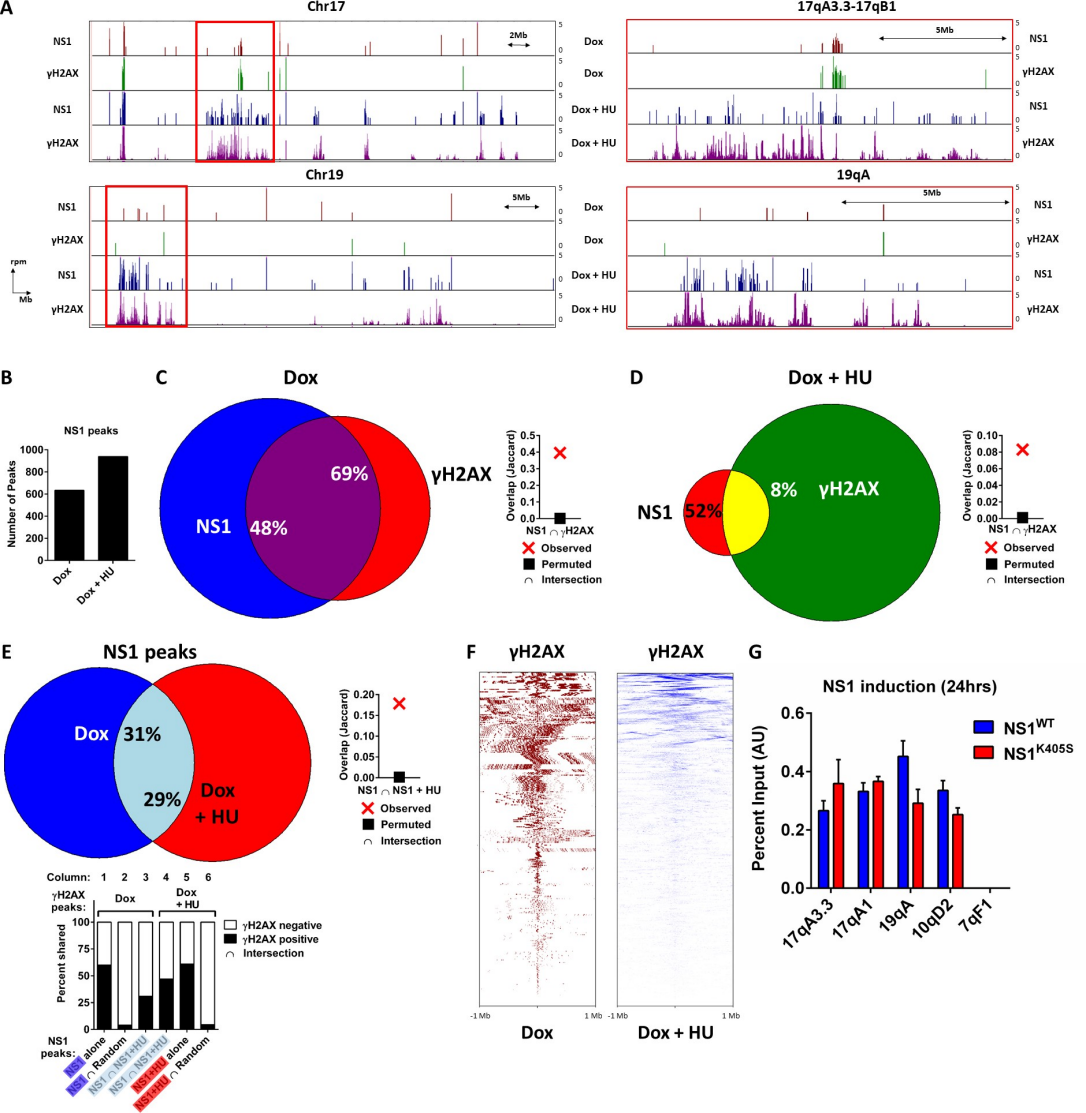
Analysis of NS1 localization and relocalization to DDR sites at the genomic level. (A) Representative NS1 and γH2AX ChIP-seq data on mouse chromosome 17 (top) and chromosome 19 (bottom) in murine A9 fibroblasts inducibly expressing NS1 for 24 hours. Cells were pulsed with 1 mM Hydroxyurea for 16 hours starting at 8 hours post-expression in the bottom 2 panels (histograms in blue and purple). Y-axis represents quantile normalized reads per million values of ChIP-seq reads for each sample. The left panels represent whole chromosome views of Chr17 and Chr19. The right panels represent zoomed-in views of the respective regions indicated by red rectangle on the left. (B) The total number of called NS1 peaks which were shared between two biological replicates of NS1 ChIP-seq experiments were compared between doxycycline induction (left) and HU pulse during doxycycline induction (right) in A9 cells containing an integrated NS1-pINDUCER20 cassette. (C,D) Venn diagrams representing whole-genome comparison of the overlap of NS1 and γH2AX ChIP-seq peaks in A9 cells expressing NS1 alone (C, left panel) and NS1 followed by HU pulse (D, left panel). The statistical significance of the overlap is shown via Jaccard analysis in the respective right panels (labelled as “Observed”; red cross). The bioinformatically-called NS1 ChIP-seq peaks common to two independent experiments were intersected with an equivalent number of randomly generated locations on the mouse genome (labelled as “Permuted”; black square). (E) The overlap of NS1 peaks throughout the genome in A9 cells expressing ectopic NS1 in the absence or presence of HU is shown via Venn diagram (E, top left) with the statistical significance of the overlap calculated by Jaccard analysis (E, top right). The intersection of the NS1 peaks with respective γH2AX peaks from two independent experiments were quantified and presented in the bar graphs (E, bottom). The subsets of NS1 peaks from the Venn diagram were represented according to the corresponding highlighted colors in the bottom panel of E. White fractions of the bars represent γH2AX-negative fraction and black bars represent γH2AX -positive fraction of identified NS1 peaks. (F) The relative position of NS1 ChIP-seq peaks with-respect-to γH2AX peaks 1 Mb upstream and downstream in the absence of HU (red; left) and the presence of HU (blue; right) were visualized using deepTools bioinformatic resource on the Galaxy project platform [63,67]. (G) Focused NS1 ChIP-qPCR assays were performed at a subset of cellular sites identified by ChIP-seq in cells inducibly expressing wild-type NS1 (NS1^WT^, blue bars), and the dimerization mutant of NS1 (NS1^K405S^, red bars) for 24 hours. Data is presented as mean ± SEM of percent input pulldown efficiencies across 2 independent experiments.

As shown for representative samples on mouse chromosomes 17 and 19, where the MVM genome was previously found to localize within megabase-sized domains during early stage of infection ([21] and described below), NS1 binds to distinct sites on Chr17 and Chr19 (red histogram), and colocalization of NS1 with γH2AX (green histogram) is evident when viewed both from the whole-chromosome view (Fig 2A left panels), as well as in magnified finer detail (Fig 2A right panels). Induction of DNA damage in these cells via an HU pulse led to the spread of γH2AX over wide areas (purple histogram and, cf. Fig 1D, panel 3), as previously characterized by others for γH2AX induction [26,31], and consistent with previously published findings during MVM infection [21]. NS1 broadly relocalized to the γH2AX domains in both analyses (blue histogram).

When analyzed on a genome-wide basis (results for individual chromosomes comprising the entire mouse genome are shown in supplemental S1 Fig), we observed that, in the presence of 1 mM HU pulsed for 16 hours to induce DNA damage, treated cells possessed approximately 50% more NS1-associated peaks relative to untreated A9 cells (Fig 2B, 635 versus 940 shared peaks obtained from the intersection of 2 independent experiments). This suggested there was increased migration of NS1 to cellular genomic sites upon HU treatment. More detailed analysis of genome-wide data from 2 separate experiments were consistent, and showed, that in the absence of HU treatment, approximately 48% of the NS1 positive sites were shared with γH2AX (Fig 2C, left). The statistical significance of this intersection was evaluated using Jaccard analysis in which the observed NS1 peaks were intersected with a randomly computed set of sites on the mouse genome containing the same number of equally sized peaks as control (Fig 2C, right, compare red cross with black square respectively). Reciprocally, 69% of γH2AX-bound regions in these cells contained NS1 (Fig 2C, left), suggesting that the majority (more than two-thirds) of γH2AX sites in non-treated cells, presumably generated by DNA damage in their vicinity, could serve as substrates for NS1 localization. Upon HU induction of additional DNA breaks during doxycycline-induced NS1 expression, we observed the formation of broad γH2AX peaks on chromosomes 2–8, 15–17 and 19 (supplemental S1 Fig). Quantification of the extent of NS1 interaction with γH2AX regions throughout the genome revealed that approximately 52% of NS1 peaks were shared with γH2AX marked regions (Fig 2D, left). This colocalization was statistically significant as evaluated by similar Jaccard analysis (Fig 2D, right). Importantly, since in the presence of HU approximately 50% more of NS1 protein targets the genome (Fig 2B), this again confirmed a significant migration of NS1 to sites of DNA damage, which corresponded in this case with only 8% of the total γH2AX ChIP-seq sites (Fig 2D, left). Interestingly, since approximately half of NS1 localized to γH2AX in the presence or absence of HU, an approximate maximum of 50% of NS1 molecules were apparently available to interact with the cellular genome.

Approximately 30% of NS1 peaks were conserved between non-HU-treated cells induced to express ectopic NS1, and those expressing NS1 in the presence of HU (Fig 2E top left; light blue). This overlap was statistically significant, as calculated by Jaccard analysis (Fig 2E, top right). Of these overlapping NS1 sites (represented by light blue region in the Venn diagram in Fig 2E), 31% colocalized with endogenous γH2AX sites (Fig 2E bottom, column 3). Co-localization to HU-induced γH2AX sites increased to 47% (Fig 2E bottom, column 4, intersection of 2 independent experiments), further demonstrating NS1 relocalization to these sites was increased upon HU treatment. Of the non-overlapping NS1 sites (represented by dark blue and red regions in the Venn diagram in Fig 2E), approximately 60% still overlapped with γH2AX, in both untreated and HU treated cells (Fig 2E bottom, columns 1 and 5, respectively), somewhat more, but similar to results in 2C and 2D. Considering that approximately 50% more NS1 interacted with the genome following HU induction (Fig 2B), these results also indicated that HU-induced sites on the genome became targeted by NS1. These intersections were statistically significant, as a randomly generated library of genomic binding sites was γH2AX-positive for less than 5% of the common sites which intersected with NS1 peaks in the presence or absence or HU (Fig 2E bottom, columns 2 and 6). When NS1 binding relative to γH2AX peaks was visualized throughout the genome (as shown in the heat maps in Fig 2F), most γH2AX peaks in untreated cells contained NS1 bound regions within their 2 Mb vicinity (red; left). However, in the presence of HU (blue; right) a smaller number of NS1 peaks were within this vicinity. This was consistent with the NS1 occupancy change from 69% in the absence of HU to 8% in its presence, as shown in Fig 2D and 2E. These heatmaps suggested further that even though all NS1 peaks did not colocalize with γH2AX peaks, the majority fell within the 2 megabase window encompassing γH2AX. Identified NS1 peaks did not correlate with published murine ChIP-seq peaks for active histone modifications H3-acetylated lysine 27 (H3K27ac, [32]), or H3-acetylated lysine 9 (H3K9ac, [33]), (S2 Fig), in spite of previous qualitative observations that during MVM infection NS1-bound regions correlated with sites of transcriptionally active Type A chromatin marks [21,34]. This suggested that the replicating viral genome might aid in aspects of virus localization that are unknown, and possibly independent of NS1.

To confirm the results described above showing that localization of NS1 to cellular sites of DNA damage was independent of its ability to directly bind DNA, we selected a subset of strongly binding NS1 peaks to assay by ChIP-qPCR in A9 cells inducibly expressing either wild-type NS1 (NS1^WT^), or the non-DNA binding K405S mutant (NS1^K405S^, described above) with doxycycline. As shown in Fig 2G, NS1 pulldown efficiencies at these sites for both NS1^WT^ and NS1^K405S^ were similar, confirming that the association of NS1 with cellular DDR sites was independent of its ability to bind DNA (as shown in Fig 1B above). These assays further suggested that components present at sites of cellular DDRs, rather than NS1’s cognate DNA binding element, likely served as mediators for NS1 binding.

### Ectopically expressed NS1 localizes with Virus Associated Domains identified during infection

**Fig 3:**
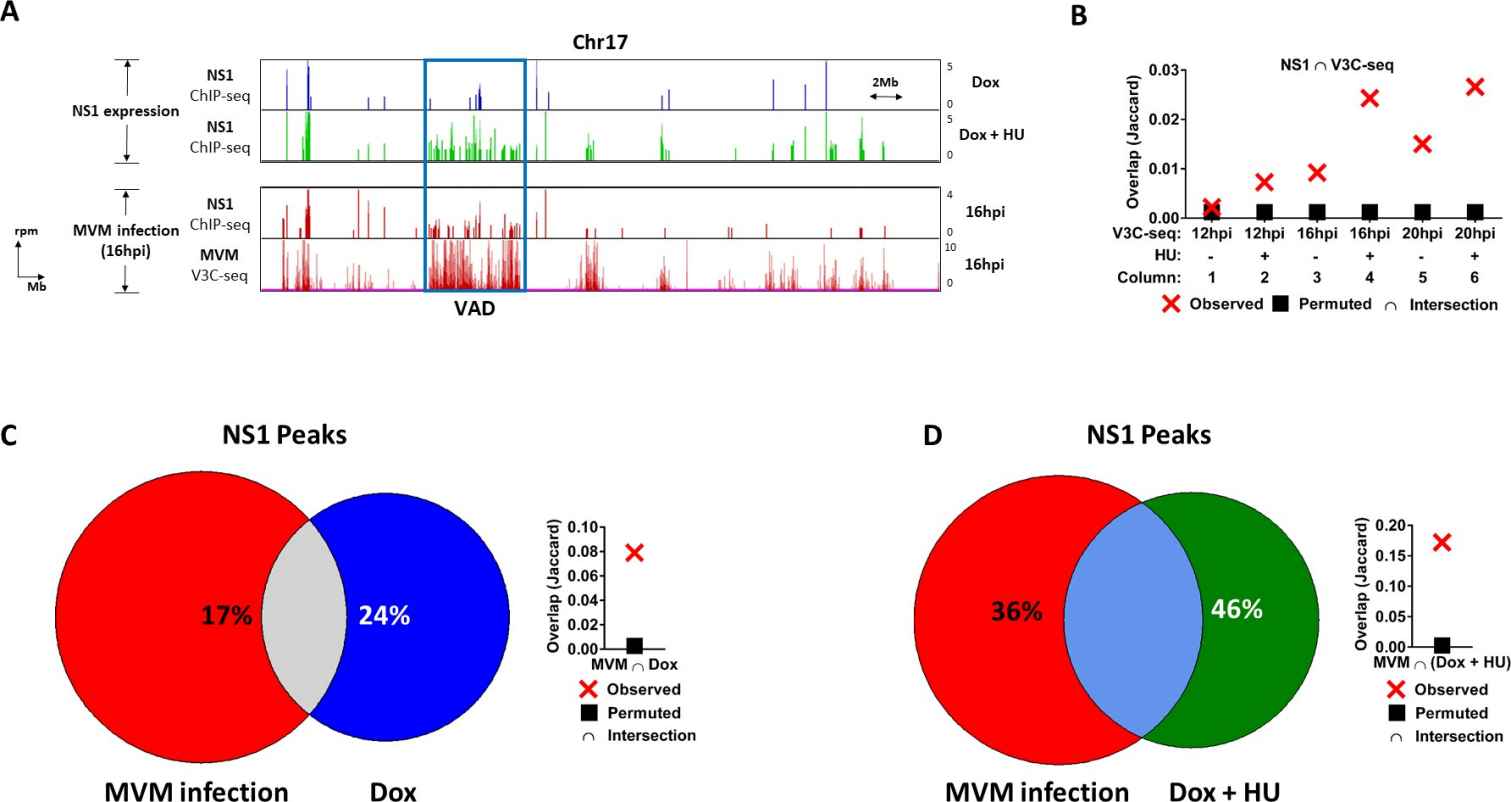
Ectopically expressed NS1 localizes with Virus Associated Domains identified during infection. (A) Representative NS1 ChIP-seq tracks on mouse chromosome 17 comparing the localization sites of ectopically expressed NS1 in the absence or presence of HU (blue and green histograms respectively) with that of NS1 during infection (red histogram; third genome browser track). The NS1 binding sites are compared with MVM localization sites previously identified using V3C-seq (red histogram; fourth genome browser track). NS1 ChIP-seq and MVM localization V3C-seq were carried out at 16 hpi and have been described previously [21]. The resulting Virus Associated Domain (VAD) has been demarcated in blue rectangle. (B) The NS1 ChIP-seq peaks identified in the presence and absence of HU were compared with MVM genome localization sites at 12 hours post-infection (hpi), 16 hpi and 20 hpi previously identified by V3C-seq [21]. The statistical significance of this overlap was determined by Jaccard analysis (red crosses), and compared with a randomly generated set of cellular sites with approximately the same number of genomic sites as the identified NS1 ChIP-seq peaks (black squares). The extent of separation between red X and black squares reflect increased correlation of ChIP-seq and V3C-seq peaks. The overlap of NS1 ChIP-seq peaks previously identified at 16 hpi of MVM infection (C, D; red, [21]) was compared with NS1 peaks identified during expression (C, blue) and expression in the presence of HU treatment (D, green). The statistical significance of the respective overlap was evaluated by Jaccard analysis (C,D; right).

In order to investigate how transiently expressed NS1’s association with the cellular genome correlated with cellular sites associated with the MVM genome during infection, we intersected NS1 ChIP-seq data gathered during doxycycline-induced NS1 expression with MVM virus associated sites (which we dubbed Virus Associated Domains, or VADs; [21]). As shown in Fig 3A, NS1 peaks which relocalized to encompass broad regions of mouse Chromosome 17 (green histogram) generally coincided with previously identified cellular VAD sites [blue rectangle encompassing NS1 ChIP-seq (red histogram; 3rd genome browser track) and MVM V3C-seq (red histogram; 4^th^ genome browser track) at 16 hpi]. The extent of correlation was therefore evaluated over time using Jaccard analysis where NS1 ChIP-seq peaks were intersected with a control library of random sites (as described above). As shown in Fig 3B, expressed-NS1 peaks did not coincide with V3C-seq peaks at 12 hpi in the absence of HU (Fig 3B, column 1). However, NS1 peaks began to correlate with MVM genome-associated regions in cells treated with HU at this time (Fig 3B, column 2). This was likely due to the broadening of NS1 interaction sites observed upon HU treatment during NS1 expression (described above). At both 16 hpi and 20 hpi, a substantial number of NS1 peaks correlated with V3C-seq sites in untreated cells, which increased further when intersected with samples that had undergone HU induction (compare columns 3 to 4 and 5 to 6 respectively).

We have previously shown that during infection at 16 hpi, 90% of NS1-associated sites—which included MVM-bound NS1 and host-chromatin-bound NS1- correlated with VADs (21). Only 17% of these peaks overlapped with NS1 sites identified in NS1-expressing cell lines in the absence of HU (Fig 3C), which approximately doubled to 36% in the presence of HU (Fig 3D). The overlap of transiently expressed NS1 peaks with NS1 during infection also approximately doubled, from 24% in the absence of HU (Fig 3C) to 46% in the presence of HU (Fig 3D). These results indicated that ectopically expressed NS1 could localize to the same shared cellular sites as NS1 associated with the MVM genome; however, the unique DDR profile induced by infection subsequently generated an additional set of target sites for NS1 on the host genome used exclusively by the virus.

### NS1 directs the localization of MVM genome to cellular DDR sites

**Fig 4:**
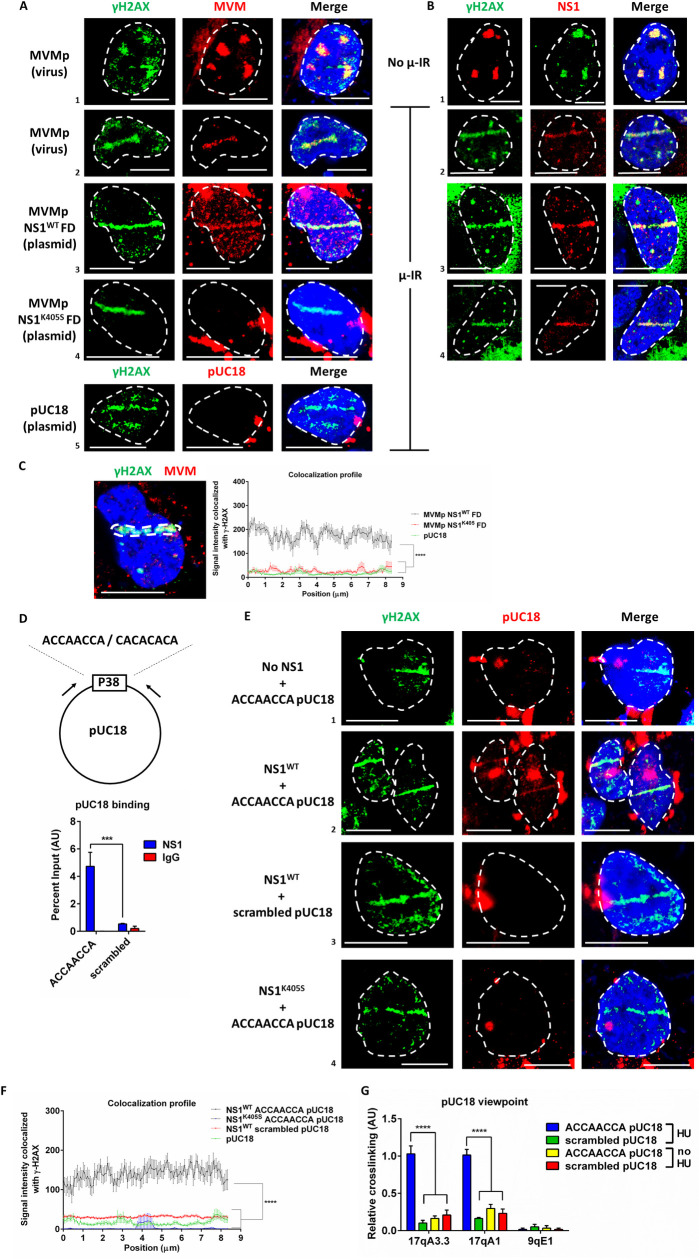
NS1 directs the localization of MVM genome to cellular DDR sites. (A) Representative immuno-FISH data of U2OS cells infected with MVMp virus for 16 hours (panels 1,2) and MVM infectious clone lacking the replication origin (MVMp FD, panels 3,4) or pUC18 vector (panel 5) transfected for 36 hours. DNA damage was induced using laser micro-irradiation (panels 2–5) and monitored by γH2AX staining (green). The MVM genome or pUC18 DNA were visualized by AF-555 labelled FISH probes (red). The white bar in the figure inset represents 10 microns. (B) The corresponding NS1 localization during infection (panels 1,2) and MVMp FD transfection (panels 3,4) were monitored using immunofluorescence assays for NS1 (red). DNA damage was induced using laser micro-irradiation (panels 2–4) and monitored by γH2AX staining (green). The white bar in the figure inset represents 10 microns. Data is representative of 3 independent experiments, each imaging at least five fields of view containing 4–5 nuclei. (C) Schematic of the selection of laser-micro-irradiated ROI (left) for the quantification of Immuno-FISH assays in multiple nuclei (right). The average FISH signal intensity of AF-555 labelled MVMp and pUC18 (red) over the laser micro-irradiated sites (green) were calculated in over 10 nuclei in the indicated conditions in Fig 4A. Data is presented as mean ± SEM of signal intensity at 72 nm intervals along ROIs that are 8.4 μm in length. Statistical analysis was performed by One-way ANOVA, multiple comparisons test. Statistical significance is designated by **** < 0.0001 for all samples relative to MVMp NS1^WT^ FD. (D) Schematic of ACCAACCA consensus site and scrambled sequence (CACACACA) on MVMP38 with 100 bp of the genome sequence on either side, which were cloned into the HindIII site of the pUC18 vector. NS1 binding to this site on the pUC18 vector was monitored by NS1 ChIP-qPCR assay (bottom), with the qPCR primers located on the pUC18 backbone flanking the insert site (black arrows). NS1 expression was induced with doxycycline for 16 hours in stable A9 cells described above before transfecting with the modified pUC18 vectors for 8 hours, followed by ChIP assays at 24 hours post-induction. Data is represented as mean ± SEM for percent input of NS1 pulldowns in three independent experiments. Statistical significance was determined by unpaired t-test. Statistical significance is shown by ***, which reflects p < 0.05. (E) Immuno-FISH assays of U2OS cells transiently expressing wild-type (NS1^WT^, panels 2,3) or dimerization mutant (NS1^K405S^, panel 4) variants of NS1, transfected with the ACCAACCA–pUC18 (panels 1,2,4) or scrambled–pUC18 (panel 3) vectors for 16 hours and damaged by laser micro-irradiation which were monitored by γH2AX staining (left panel). Plasmid localization was monitored by AF-555 labelled probe (middle panels) and their colocalization was visualized in the right panel (labelled “Merge”). (F) Quantification of the average FISH signal intensity of AF-555 labelled pUC18 (red) over the laser micro-irradiated sites (green) calculated in over 10 nuclei in the indicated conditions in Fig 4E. Data is presented as mean ± SEM of signal intensity at 72 nm intervals along ROIs that are 8.4 μm in length. Statistical analysis was performed by One-way ANOVA- multiple comparisons test. Statistical significance is designated by **** < 0.0001 for all the samples relative to NS1^WT^ ACCAACCA-pUC18. (G) ChIP-loop assays with pUC18 viewpoint in NS1-expressing A9 fibroblasts (induced for 24 hours with doxycycline) transfected with the ACCAACCA–pUC18 / scrambled–pUC18 plasmids at 12 hours post-induction followed by DDR induction with hydroxyurea at 18 hours post-induction. Chromatin was processed using standard 3C methods before performing NS1-ChIP, and intramolecular ligation was subsequently performed. The localization of the pUC18 vector with cellular DDR sites (identified by γH2AX ChIP-seq, Fig 2) was assayed by Taqman qPCR where the probe viewpoint is complementary to the plasmid genome while the assay sites are on the mouse genome are at chromosome 17qA1, 17qA3.3 and the previously identified gene desert site 9qE1 [21]. Statistical analysis was performed by one-way ANOVA- multiple comparisons test. Statistical significance is designated by **** < 0.0001.

As a prelude to further analysis of viral genome re-localization, we initially optimized 3-color Immuno-FISH assays in MVM infected U2OS cells. Following staining samples with γH2AX to identify the sites of cellular DNA damage, we monitored co-localization of the viral genome using Alexa-Fluor-555-labelled MVM (Fig 4A) and anti-NS1 (Fig 4B). As shown in Fig 4A (panel 1) the viral genome and γH2AX colocalized in MVM infected cells at nuclear sites previously identified as APAR bodies (identified by NS1 staining in Fig 4B, panel 1). Upon induction of DNA damage using laser micro-irradiation, the MVM genome relocalized to induced DDR sites (Fig 4A, panel 2). Following transfection in U2OS cells, we also found that a non-replicating MVM genomic plasmid (labelled as MVMp NS1^WT^ FD), which includes its multiple genomic NS1 binding sites, could similarly localize to micro-irradiated nuclear sites (Fig 4A, panel 3). Concurrently, the wild-type NS1 protein expressed by this vector also colocalized to the laser micro-irradiated sites (Fig 4B, panel 3). Strikingly, a transfected MVMp FD genome which expressed the NS1^K405S^ mutant that lacks the ability to dimerize and bind to its cognate consensus sequence (described above and labelled here as MVMp NS1^K405S^ FD) did not relocalize to the laser micro-irradiated stripe (Fig 4A, panel 4). However, the expressed mutant NS1^K405S^ protein localized to the laser stripe (Fig 4B, panel 4, and as seen previously). Similarly, an unrelated pUC18 vector did not localize to the stripe (Fig 4A, panel 5). Quantification of the average profile of MVM DNA which colocalized with laser micro-irradiated regions (schematic shown in Fig 4C, left) in multiple nuclei confirmed that the MVMp NS1^WT^ FD plasmid localized with γH2AX significantly better than the MVMp NS1^K405S^ FD plasmid, which remained distinct from these regions, similar to the pUC18 plasmid background (Fig 4C, right). These findings suggested that while NS1 did not require its DNA binding ability to localize to DDR sites, localization of the MVM genome to sites of DNA damage could occur only when wild type NS1, but not its DNA binding mutant, was expressed.

To test whether NS1 might be capable of transporting a heterologous DNA molecule to cellular sites of DNA damage, we inserted the P38 promoter region of MVM, which contains a potent NS1 binding ACCAACCA site, into the pUC18 vector (Fig 4D, top). Control vectors were generated containing the same insert except that the NS1 binding sequence was scrambled to CACACACA (hereafter labelled as “scrambled-pUC18”). Focused NS1 ChIP-qPCR assays showed that NS1 bound to the pUC18 vector containing ACCAACCA consensus sequence at nine-fold higher efficiency than the vector containing scrambled consensus sequence following their transfection in inducible NS1-expressing stable cells (Fig 4D, bottom).

In the absence of NS1, neither the pUC18 vector alone (Fig 4A, panel 5), nor the ACCAACCA-pUC18 vector (Fig 4E, panel 1), localized to the laser micro-irradiated sites. Expression of NS1^WT^ in these cells prior to laser micro-irradiation led to the re-localization of ACCAACCA-pUC18 to the induced laser stripe (Fig 4E, panel 2). However, neither the scrambled-pUC18 vector in the presence of NS1^WT^, nor the ACCAACCA-pUC18 vector in the presence of the non-DNA-binding NS1^K405S^ protein, localized to laser micro-irradiated sites (Fig 4E, panels 3 and 4, respectively). These results indicated that NS1 binding to its cognate binding sequence was essential for localization of the plasmid to induced DDR sites. Quantification of the immuno-FISH studies over multiple micro-irradiated nuclei supported these observations (Fig 4F). These findings suggested that when bound, NS1 can translocate a heterologous DNA molecule to cellular sites of DNA damage.

As a complementary approach, we performed ChIP-loop experiments, which combine chromatin immunoprecipitation with chromosome conformation capture [35]. In this case, we first identified robust HU-induced γH2AX sites by ChIP-seq (Fig 2) to monitor NS1-bound modified pUC plasmid localization. If the heterologous molecule was recruited to these HU-induced DDR sites by NS1, then these assays should yield hybrid junctions between pUC18 and the cellular DDR regions of interest after intramolecular ligations are performed. As shown in Fig 4G, in the presence of HU, the NS1-bound ACCAACCA-pUC18 molecule formed novel ligation junctions with 17qA3.3 and 17qA1 respectively, two regions on the mouse genome containing robust HU-induced γH2AX peaks identified in Fig 2. In the absence of HU (when DNA damage was not detected at these sites), or when the pUC18 vector containing scrambled NS1 binding sites was used, the frequency of these novel ligation junctions was significantly diminished (Fig 4G)—suggesting that these heterologous molecules localized less efficiently to these regions. Additionally, even in the presence of HU, the NS1-bound ACCAACCA-pUC18 molecule did not form detectable hybrid junctions at a gene desert region of the genome that is devoid of DNA damage following HU treatment, previously identified on chromosome 9 (9qE1, [21]), suggesting that the localization occurred primarily at HU-induced regions. These findings independently confirmed our microscopic observations that NS1 bound to a heterologous DNA molecule was necessary to translocate it to a cellular DDR site.

## Discussion

We have previously shown that MVM establishes replication centers at cellular sites of DNA damage which associate with γH2AX [21]. To begin to understand how such centers are established and maintained during ongoing viral replication, we examined how individually expressed MVM NS1 localizes to cellular sites of DNA damage. We have found that NS1 localizes to sites of cellular DNA damage in a manner independent of its ability to bind DNA. Many of these sites overlap with sites of viral association during infection. Further, the MVM genome, as well as a heterologous DNA molecule containing an NS1 binding site could be localized to sites of cellular DNA damage in the presence of wild-type, but not DNA binding mutant of NS1. These results suggested that NS1 may play a role in localizing the MVM genome to sites of DNA damage to facilitate ongoing infection.

During ectopic expression in the absence of replicating viral genome substrates, non-MVM-bound NS1 relocated to chemical- and radiation-induced nuclear DNA breaks. This was observed first in microscopic assays. Genome-wide ChIP-seq data then confirmed and expanded on these observations. In untreated cells, NS1 was found to associate with sites of DNA damage bound by γH2AX; however, when cells were treated with HU, a significant portion of NS1 migrated to the additional sites of damage that was induced. As NS1 possesses both helicase and nickase functions [36,37], it is also possible that ectopic NS1 causes genome instability by directly disrupting the cellular DDR machinery, or indirectly, by causing cellular toxicity [38], either of which would result in increased γH2AX foci. However, these possibilities remain to be mechanistically tested. Importantly however, migration to cellular sites of DNA damage did not require NS1’s ability to bind DNA. NS1 might be targeted to cellular DDR sites in a DNA-binding-independent manner through its interaction with host proteins, which are yet to be determined. In this regard, multiple SQ/TQ motifs on NS1 could serve as phosphorylation targets for ATM kinase, which is also active at the DNA break sites [39]. Additionally, Casein Kinase 2 alpha (CK2α), which mediates the recruitment of the DDR adaptor protein MDC1 to DNA break sites marked by the MRN complex, also interacts with NS1 [40,41]. While this is yet to be determined, the interaction between NS1 and factors present at DDR sites are likely important for NS1 localization.

It remains unclear specifically if the viral genome localizes to cellular DDR sites at the earliest stages of infection, before NS1 can be expressed. If it does, it is possible that the incoming capsid may play a role. In addition, cis-elements on MVM, bound by proteins that can localize to cellular DDR sites, could also impact genome localization. Factors including CTCF and cohesin, which contain binding sites on MVM and to cellular promoters respectively, and are known to localize to cellular sites of DNA damage in the nucleus, are candidates for such a role [42–44]. The DDR adaptor molecule MDC1, which dimerizes to bridge the DNA-break sensing MRN complex and γH2AX chromatin marks, remains distinct from MVM APAR bodies during infection [20]. If the phosphorylated isoform of MDC1 at threonine-4 (MDC1PT4) associated with its dimerization [40,45,46] also colocalizes with APAR bodies, it could suggest that MDC1 contributes to genome localization.

Our observation of NS1 translocating a bound DNA molecule to a cellular DDR site suggests that NS1 transports MVM genomes to sites of DNA damage as well as to additional sites of genomic DNA breaks, as the genome amplifies. The Large T antigen (LT) protein of the DNA tumor virus SV40 induces cellular DNA damage while also associating with the viral genome at 3 sites [47,48]. The connecting link between LT and the cellular DDR pathways are the proteins BUB1 and NBS1, which interact with LT on the SV40 genome [48–50]. The HPV genome associates with cellular fragile sites through interaction between the E2 protein and BRD4, a chromatin-targeting cellular protein at sites called PEB-BLOCs which also regulates PCNA loading on eukaryotic replication forks [51,52]. The transport of E2-bound HPV genome to PEB-BLOCs before being tethered to the host genome by BRD4-mediated bridging seems to present a potential mechanism by which papillomaviruses localize to their appropriate nuclear sites. Interestingly, the HPV E2- associated genomic sites are enriched in active chromatin [51] similar to EBNA3A of EBV, which also binds to the cellular genome at strong enhancers and poised promoters [53]. Although MVM-genome associated VAD sites are enriched in active chromatin (mostly Type A; [21,34]), ectopic NS1 colocalizes with limited regions of active host chromatin, instead showing a preference for γH2AX (Fig 2F). Since only a portion of VAD sites are shared by individually expressing NS1, even if viral proteins transport the viral genome to DNA break sites, it is possible that the genome amplifies to occupy regions of active chromatin induced during infection. How small DNA viruses establish their ongoing replication centers is a fundamental aspect of their life cycle, and for the parvovirus MVM, the large non-structural protein NS1 likely participates in this process.

## Materials and methods

### Contact for reagent and resource sharing

Further information and requests for resources and reagents should be directed to and will be fulfilled by Kinjal Majumder (km3k5@missouri.edu) and David J. Pintel (pinteld@missouri.edu).

### Cell lines, virus and virus infections

Male murine A9 and female human U2OS cells were propagated in 10 percent Serum Plus (Sigma Aldrich) containing DMEM media (Gibco) supplemented with Gentamicin at 37 degrees Celsius and 5 percent carbon dioxide. A9 and U2OS cells were used to demonstrate the generalizability of NS1 localization to cellular sites of DNA damage. Cell lines are routinely authenticated for mycoplasma contamination, and background levels of DNA damage detected by γH2AX staining. As A9 cells have smaller nuclei and higher background γH2AX levels relative to U2OS, imaging-based studies of NS1 localization to cellular DDR sites can be difficult to discern microscopically using this system. Therefore, U2OS cells were predominantly used for the laser microirradiation and imaging assays. Wild-type MVMp and the 1989 mutant of MVM (MVMp^ΔNS2^) were produced as previously described [15,24] and genome copies were quantified by Southern blotting [54]. MVM infection was carried out at a Multiplicity of Infection (MOI) of 10. Lentivirus constructs designed to inducibly express NS1 were generated by co-transfecting equal concentrations of HIV Gag/Pol, Vesicular Stomatitis Virus glycoprotein G (VSV-G) and relevant pINDUCER20 plasmids containing NS1^WT^ and NS1^K405S^ into HEK293T cells for 48 hours [22,28]. Stable doxycycline-inducible A9 cell lines were selected with 800 μg/ml of Geneticin (Gibco). pINDUCER20 lentivirus-transformed cell lines were induced with 500 ng/ml Doxycycline hydrochloride (MP Biomedicals) for the indicated amount of time.

### Cell synchronization and drug treatments

A9 cells were parasynchronized in G0 phase of cell cycle by isoleucine deprivation for 42 hours as previously described [15]. Cells were infected with MVM upon release into complete DMEM medium (described above). The cells enter S phase approximately 12 hours post release [15,22,24]. A9 cells containing an integrated NS1-pINDUCER20 cassette were induced with 500 ng/ml Doxycycline hydrochloride (MP Biomedicals) upon release. For ChIP-seq experiments, parasynchronized A9 cells containing an integrated NS1-pINDUCER20 cassette were treated with 1 mM Hydroxyurea (Sigma Aldrich) at 8 hours post release for 16 hours as indicated in Fig 2. For immunofluorescence assays in U2OS cells expressing NS1 for 16 hours, doxorubicin hydrochloride (Sigma Aldrich) treatment was carried out at a final concentration of 200 nM and HU treatment was carried out at a final concentration of 1 mM, starting at 12 hours post-transfection for 4 hours.

### Plasmids and transfections

The NS1 ORF was cloned into the mammalian expression vectors pCDNA3.1 (Invitrogen) as previously described [55]. This vector was used as the template to generate pINDUCER20 cassettes containing the NS1 ORF. The MVM p38 site containing ACCAACCA consensus sequence, as well as the scrambled consensus sequence were cloned into the pUC18 vector using gene blocks flanked by HindIII restriction enzyme sites (Supplemental file 1) and transformed into DH5α competent cells. pINDUCER20 reagents were a gift from Guang Hu (NIH/NIEHS) [23,28]. Transient transfection of cells were performed using LipoD293 (SignaGen Laboratories).

### Laser micro-irradiation for immunofluorescence

Laser micro-irradiation was performed on 1 million A9 or U2OS cells cultured on glass bottomed dishes (MatTek Corp.) infected with MVM at an MOI of 10 for 18 hours, transfected with 1 microgram of NS1-expression vectors for 16 hours or induced with Doxycycline for 16 hours. Cells were sensitized with 3 μl of Hoechst dye (ThermoFisher Scientific) 5 minutes prior to micro-irradiation. Samples were irradiated using a Leica TCP SP8 confocal microscope using 40X oil objective and 3X digital zoom with a 405 nm laser using 25 percent power at 40 Hz frequency for 1 frame per field of view. Regions of interest (ROIs) were selected across the nucleus such that they did not traverse the nuclear membrane. Samples were processed for immunofluorescence imaging (described below) immediately after micro-irradiation.

### Immunofluorescence assays

A9 and U2OS cells were pre-extracted with CSK Buffer (10 mM PIPES pH 6.8, 100 mM Sodium Chloride, 300 mM Sucrose, 1 mM EGTA, 1 mM Magnesium Chloride) and CSK with 0.5% Triton X-100 for 3 minutes each before fixation with 4 percent paraformaldehyde for 10 minutes at room temperature. Cells were washed in PBS before being permeabilized with 0.5% Triton X-100 in PBS for 10 minutes at room temperature. Samples were blocked with 3% BSA in PBS for 1 hour, incubated with the primary antibody diluted in 3% BSA solution for 1 hour, washed in PBS and incubated with secondary antibody (tagged with Alexa Fluor fluorophores) for 1 hour. Samples were washed in PBS and mounted on slides with DAPI Fluoromount (Southern Biotech).

### Immuno-FISH assays

U2OS cells were grown on glass bottomed dishes for laser micro-irradiation induced DNA damage after sensitization with 3 μl Hoechst 3342 (3X digital zoom; 33Hz; 15% power). Samples were pre-extracted in CSK (recipe described above) for 3 minutes, followed by CSK with 0.5% Triton X-100 for 3 minutes before washing with PBS and fixing with 4% PFA in PBS for 10 minutes. Calls were washed with PBS before being permeabilized in 0.5% Triton X-100 in PBS for 10 minutes at room temperature. Samples were treated with 2 μl RNase A in PBS for 1 hour at 37 degrees Celsius. Samples were denatured in 70% Formamide solution (70% Formamide, 2X SSC) for 3 minutes at 77.8 degrees Celsius, 50% Formamide solution (50% Formamide, 2X SSC) for 1 minute, before adding the denatured FISH probe in Hybridization mixture (1 ul probe, 25 ul Hybridization buffer, 15 μl sonicated salmon sperm DNA), sealed with parafilm and incubated at 37 degrees Celsius for 30 minutes. Samples were washed 3 times with 2X SSC/0.1% Triton X-100 at 37 degrees Celsius for 5 minutes each, followed by 3 washes in 2X SSC at 37 degrees Celsius for 5 minutes each. Samples were blocked in 3% BSA in PBS for 1 hour at room temperature in the dark, and processed for immunofluorescence as described above.

DNA probe labelling for Immuno-FISH assays were carried out using the FISH Tag DNA Multicolor Kit from ThermoFisher Scientific according to manufacturer’s protocol.

The intensity of FISH signal over the laser micro-irradiated region was quantified using the plotprofile tool on ImageJ [56]. Briefly, 8.4 μm ROIs were selected where γH2AX resulting from laser stripe and FISH signals coincided. The FISH signal intensity was calculated in 72 nm intervals along the ROI for at least 10 nuclei, and averaged using Graphpad Prism.

### Antibodies

Mouse anti-γH2AX (EMD Millipore; 05–636), rabbit anti-γH2AX (Abcam; ab11174), rabbit anti-NR5A2 (Abcam; ab189876), rabbit anti-FOXP1 (Cell Signaling; 2005S), anti-mouse AF488 secondary (Life Technologies; A11029), anti-rabbit AF488 secondary (Life Technologies; A11034), anti-mouse AF568 secondary (Life Technologies; A11031), anti-rabbit AF568 secondary (Life Technologies; A11036). The mouse anti-NS1 monoclonal antibody has been previously generated and validated by the Pintel lab [21].

### Chromatin Immunoprecipitation followed by sequencing (ChIP-seq)

A9 cells were crosslinked with 1% formaldehyde for 10 minutes on a rocker at room temperature before being quenched with glycine (0.125 M) for 5 minutes on ice. Cells were collected in cold PBS followed by lysis in ChIP lysis buffer (1% SDS, 10 mM EDTA, 50 mM Tris-HCl, pH 8, protease inhibitors). The whole-cell lysates were sonicated using a Diagenode Bioruptor for 60 cycles (30 seconds on and 30 seconds off). The samples were centrifuged at 4 degrees Celsius for 10 minutes before the lysate was added to suspensions of antibodies and Protein A Dynabeads (Thermo Scientific). The samples were incubated by rotation at 4 degrees Celsius overnight before being washed with Low Salt Wash (0.1% SDS, 1% Triton X-100, 2 mM EDTA, 20 mM Tris-HCl pH 8, 150 mM NaCl), High Salt Wash (0.1% SDS, 1% Triton X-100, 2 mM EDTA, 20 mM Tris-HCl pH 8, 500 mM NaCl), Lithium Chloride Wash (0.25 M LiCl, 1% NP-40, 1% Deoxycholate, 1 mM EDTA, 10 mM Tris-HCl pH 8) and twice with TE wash buffers before being eluted in SDS elution buffer (1% SDS, 0.1 M Sodium Bicarbonate). Following elution, the chromatin-antibody complexes were reverse-crosslinked with 100 mM sodium chloride heated at 65 degrees Celsius and Proteinase K for 2 hours. The eluted DNA was purified using a Qiagen PCR purification kit and samples were eluted in 50 ul of Buffer EB (Qiagen).

Sequencing libraries were generated from ChIP DNA using the NEBNext Ultra II Library Prep Kit for Illumina, and the sonication quality was verified using Agilent Bioanalyzer. For ChIP-seq assays, all nine samples were pooled and sequenced on an Illumina Next Seq 500 instrument using 75 base-pair Single End Sequencing.

ChIP-seq samples were aligned to the mouse genome (build mm10) using Bowtie2 [57]. The resultant SAM files were converted to BAM files using Samtools [58]. BED files were generated using BEDtools [59]. Peaks were called with EPIC analysis software using the SICER algorithm [60–62] according to default parameters. Called peaks that were shared between 2 independent biological replicates were identified using BEDtools software [59]. In order to compare the magnitudes of ChIP-seq peaks between different treatment conditions, rpm values were calculated (using Galaxy project, [63]) on the bedgraph files generated from EPIC, and were quantile normalized using preprocessCore package on RStudio [64]. Statistical significance of overlapping ChIP-seq peaks were performed using the Jaccard function on BEDtools software. The Jaccard analysis represents the ratio of the intersection of two sets (in this case, called ChIP-seq peaks) to that of the union of the same sets. The resultant overlap is represented as a decimal between 0 (reflecting no overlap) and 1 (complete overlap). “Random” BED files were permuted using BEDtools, such that they contained comparable number of peaks of similar size as determined by NS1 ChIP-seq assays, ensuring that the size of the sets being compared were similar, which were subsequently sorted before calculation by Jaccard analysis. Bioinformatic codes used have been provided in S2 Table.

### ChIP-loop assays

2 million A9 cells modified to inducibly express NS1 using the pINDUCER lentiviral system (22, 28) were grown in 100 mm tissue culture plates. Cells were induced with doxycycline for 24 hours. Cells were transfected with 0.5 μg of modified pUC18 plasmid (described above) using LipoD293 transfection reagent (Signagen) at 12 hours post-induction. Cells were pulsed with 1mM Hydroxyurea (Sigma) at 18 hours post-induction and harvested for ChIP-loop at 24 hours. Cells were crosslinked in 2% Formaldehyde for 10 minutes at room temperature by shaking, followed by quenching of the crosslinks with 0.125M Glycine for 5 minutes at room temperature on the rocker. The cells were scraped and collected into tubes, washed with PBS and lysed with 500 μl 3C lysis buffer (10 mM Tris-HCl pH 7.5, 10 mM NaCl, 0.2% NP-40, [21,65]) supplemented with 1X protease inhibitor (MedChemExpress) for 10 minutes on ice. Cells were spun down, aspirated and the resulting nuclei were resuspended in 500 μl of 1.2X NEB Buffer 2. Nuclei were permeabilized in 0.3% SDS for 30 minutes at 37 degrees C in a shaker. The permeabilization agent was sequestered with 2% Triton X-100 for 30 minutes at 37 degrees C in a shaker. The chromatin was digested with 400U HindIII overnight by shaking at 37 degrees Celsius, followed by 300U HindIII for 3 hours next day. Simultaneously, 40 μl of Protein A Dynabeads (Thermo Scientific) were washed 3 times in 1 ml of 0.02% Tween 20 in PBS for 3 minutes on a rotator at 4 degrees Celsius. The Dynabeads were incubated with 2 μg of anti-NS1 protein for 4 hours on a rotator at 4 degrees Celsius. The digested 3C chromatin was inactivated in 56 degrees Celsius for 15 minutes before the bead-antibody mixture were combined, and incubated overnight at 4 degrees Celsius on a rotator. Samples were washed twice in 1% Triton X-100 in PBS and twice in ChIP-loop wash buffer (10 mM Tris-HCl, pH 8.0, 0.25 M LiCl, 0.5% NP-40, 0.5% DOC, 1 mM EDTA) before resuspending the beads in 100 μl 1X T4 DNA Ligase Reaction Buffer (NEB). Samples were ligated overnight with 100U of T4 DNA Ligase (NEB) at room temperature on a rotator. DNA was eluted with 50 μl of ChIP-Elution Buffer (described above) twice at 56 degrees Celsius. Crosslinks were reversed with 10 μl of 5M NaCl and 5 μl of Proteinase K at 56 degrees Celsius overnight. DNA was purified using a PCR Purification Kit (Qiagen) and samples were eluted twice in 50 μl of Buffer EB (Qiagen). DNA ligation junctions were analyzed by Taqman qPCR, with the viewpoint probe on the modified pUC18 vector containing the P38 sequence and capture primers described in S1 Table.

### Quantification and statistical analysis

Statistical analysis was performed using GraphPad Prism (GraphPad Software), and BEDtools 2.26.0 [59]. Quantile normalization of ChIP-seq data was carried out on RStudio using the preprocessCore package [64].

## Supporting information

S1 Fig Analysis of NS1 localization and relocalization to DDR sites throughout the mouse genome. Representative NS1 and γH2AX ChIP-seq data throughout the mouse genome in A9 fibroblasts inducibly expressing NS1 for 24 hours (top 2 panels, in red and green histograms), and in cells pulsed with 1 mM Hydroxyurea for 16 hours starting at 8 hours post-expression (bottom 2 panels, in blue and purple histograms). Y-axis represents quantile normalized reads per million values of ChIP-seq reads for each sample. The chromosome number and scales are indicated for each chromosome throughout the mouse genome.
(TIF)

S2 Fig Comparison of NS1 binding sites with that of active chromatin marks in fibroblasts. The relative position of NS1 ChIP-seq peaks when NS1 was expressed alone (using doxycycline; top panels in red) and when expressed in the presence of HU (bottom panels in blue) were visualized 1 Mb upstream and downstream of previously published active histone modifications for H3K27ac (left; [32]) and H3K9ac (right; [33]) in 3T3 cells, using deepTools bioinformatics resource on the Galaxy project platform [63,67].
(TIF)

S1 Table Table of primer sequences used. (DOCX)

S2 Table Table of bioinformatics codes used. (DOCX)

S1 Data Spreadsheet of underlying data used. (XLSX)

## References

[1] Cotmore SF, Tattersall P. Structure and organization of the viral genome In: Kerr J, et al, editor. Parvoviruses. London, UK: Hodder Arnold; 2006 p. 73–94.

[2] Tattersall P, Ward DC. Rolling hairpin model for replication of parvovirus and linear chromosomal DNA. Nature. 1976;263:106 10.1038/263106a0 967244

[3] Cotmore SF, Tattersall P. A rolling-hairpin strategy: basic mechanisms of DNA replication in the parvoviruses In: Kerr J, et al, editor. Parvoviruses. London, UK: Hodder Arnold; 2006 p. 171–88.

[4] Naeger LK, Cater J, Pintel DJ. The small nonstructural protein (NS2) of MVM is required for efficient DNA replication and infectious virus production in a cell-type specific manner. Journal of Virology. 1990;64:6166–75. 2147041 10.1128/jvi.64.12.6166-6175.1990 PMC248791

[5] Tullis GE, Labieniec-Pintel L, Clemens KE, Pintel D. Generation and characterization of a temperature-sensitive mutation in the NS-1 gene of the autonomous parvovirus minute virus of mice. JVirol. 1988;62(8):2736–44. 2969054 10.1128/jvi.62.8.2736-2744.1988 PMC253707

[6] Cotmore SF, Tattersall P. Parvovirus diversity and DNA damage responses. Cold Spring Harb Perspect Biol. 2013;5(2). 10.1101/cshperspect.a012989 PMC3552509 23293137

[7] Bashir T, Horlein R, Rommelaere J, Willwand K. Cyclin A activates the DNA polymerase delta-dependent elongation machinery in vitro: A parvovirus replication model. Proceedings of the National Academy of Sciences. 2000;97(10):5522–7. 10.1073/pnas.090485297 PMC25861 10792046

[8] Christensen J, Cotmore SF, Tattersall P. Minute virus of mice transcriptional activator protein NS1 binds directly to the trans-activation region (tar) of the viral P38 promoter in a strictly ATP-dependent manner. Journal of Virology. 1995;69:5422–30. 10.1128/JVI.69.9.5422-5430.1995 7636987 PMC189388

[9] Willwand K, Moroianu A, Hörlein R, Stremmel W, Rommelaere J. Specific Interaction of the Nonstructural Protein NS1 of Minute Virus of Mice (MVM) With [ACCA](2) Motifs in the Centre of the Right-End MVM DNA Palindrome Induces Hairpin-Primed Viral DNA Replication Journal of General Virology. 2002;83:1659–64. 10.1099/0022-1317-83-7-1659 12075084

[10] Mouw M, Pintel D. Amino acids 16–275 of minute virus of mice NS1 include a domain that specifically binds (ACCA) 2-3-containing DNA. Virology. 1998;251:123–31. 10.1006/viro.1998.9375 9813208

[11] Cotmore SF, Nuesch JP, Tattersall P. In vitro excision and replication of 5' telomeres of minute virus of mice DNA from cloned palindromic concatemer junctions. Virology. 1992;190(1):365–77. 10.1016/0042-6822(92)91223-h 1388310

[12] Cotmore SF, Nuesch JP, Tattersall P. Asymmetric resolution of a parvovirus palindrome in vitro. J Virol. 1993;67(3):1579–89. 10.1128/JVI.67.3.1579-1589.1993 8437230 PMC237529

[13] Cotmore SF, Tattersall P. The NS-1 polypeptide of minute virus of mice is covalently attached to the 5' termini of duplex replicative-form DNA and progeny single strands. Journal of Virology. 1988;62:851–60. 10.1128/JVI.62.3.851-860.1988 3339715 PMC253642

[14] Dettwiler S, Rommelaere J, Nuesch JP. DNA unwinding functions of minute virus of mice NS1 protein are modulated specifically by the lambda isoform of protein kinase C. J Virol. 1999;73(9):7410–20. 10.1128/JVI.73.9.7410-7420.1999 10438831 PMC104268

[15] Adeyemi RO, Landry S, Davis ME, Weitzman MD, Pintel DJ. Parvovirus minute virus of mice induces a DNA damage response that facilitates viral replication. PLoS Pathog. 2010;6(10):e1001141 10.1371/journal.ppat.1001141 20949077 PMC2951379

[16] Majumder K, Etingov I, Pintel DJ. Protoparvovirus Interactions with the Cellular DNA Damage Response. Viruses. 2017;9. 10.3390/v9110323 PMC5707530 29088070

[17] Bashir T, Rommelaere J, Cziepluch C. In vivo accumulation of cyclin A and cellular replication factors in autonomous parvovirus minute virus of mice associated replication bodies. Journal of Virology. 2001;75(9):4394–8. 10.1128/JVI.75.9.4394-4398.2001 11287588 PMC114184

[18] Cziepluch C, Lampel S, Grewenig A, Grund C, Lichter P, Rommelaere J. H-1 parvovirus-associated replication bodies: a distinct virus-induced nuclear structure. J Virol. 2000;74(10):4807–15. 10.1128/jvi.74.10.4807-4815.2000 10775619 PMC112003

[19] Adeyemi RO, Pintel DJ. The ATR signaling pathway is disabled during infection with the parvovirus minute virus of mice. J Virol. 2014;88(17):10189–99. 10.1128/JVI.01412-14 24965470 PMC4136315

[20] Ruiz Z, Mihaylov IS, Cotmore SF, Tattersall P. Recruitment of DNA replication and damage response proteins to viral replication centers during infection with NS2 mutants of Minute Virus of Mice (MVM). Virology. 2011;410(2):375–84. 10.1016/j.virol.2010.12.009 21193212 PMC3072075

[21] Majumder K, Wang J, Boftsi M, Fuller MS, Rede JE, Joshi T, et al Parvovirus minute virus of mice interacts with sites of cellular DNA damage to establish and amplify its lytic infection. Elife. 2018;7. 10.7554/eLife.37750 PMC6095691 30028293

[22] Adeyemi RO, Pintel DJ. Replication of minute virus of mice in murine cells is facilitated by virally induced depletion of p21. J Virol. 2012;86(15):8328–32. 10.1128/JVI.00820-12 22623787 PMC3421664

[23] Adeyemi RO, Fuller MS, Pintel DJ. Efficient Parvovirus Replication Requires CRL4Cdt2-Targeted Depletion of p21 to Prevent Its Inhibitory Interaction with PCNA. PLoS Pathog. 2014;10(4):e1004055 10.1371/journal.ppat.1004055 24699724 PMC3974872

[24] Fuller MS, Majumder K, Pintel DJ. Minute Virus of Mice Inhibits Transcription of the Cyclin B1 Gene during Infection. Journal of Virology. 2017;91(14). 10.1128/JVI.00428-17 PMC5487563 28446681

[25] Adeyemi RO, Pintel DJ. Parvovirus-induced depletion of cyclin B1 prevents mitotic entry of infected cells. PLoS Pathog. 2014;10(1):e1003891 10.1371/journal.ppat.1003891 24415942 PMC3887112

[26] Barlow JH, Faryabi RB, Callen E, Wong N, Malhowski A, Chen HT, et al Identification of Early Replicating Fragile Sites that Contribute to Genome Instability. Cell. 2013;152:620–32. 10.1016/j.cell.2013.01.006 23352430 PMC3629730

[27] Cotmore SF, Christensen J, Nuesch JP, Tattersall P. The NS1 polypeptide of the murine parvovirus minute virus of mice binds to DNA sequences containing the motif [ACCA]2–3. J Virol. 1995;69(3):1652–60. 10.1128/JVI.69.3.1652-1660.1995 7853501 PMC188764

[28] Meerbrey KL, Hu G, Kessler JD, Roarty K, Li MZ, Fang JE, et al The pINDUCER lentiviral toolkit for inducible RNA interference in vitro and in vivo. Proc Natl Acad Sci U S A. 2011;108(9):3665–70. 10.1073/pnas.1019736108 21307310 PMC3048138

[29] Sneeden JL, Loeb LA. Mutations in the R2 subunit of ribonucleotide reductase that confer resistance to hydroxyurea J Biol Chem. 2004;279:40723–8. 10.1074/jbc.M402699200 15262976

[30] Pommier Y, Leo E, Zhang H, Marchand C. DNA topoisomerases and their poisoning by anticancer and antibacterial drugs Chem Biol. 2010;17:421–33. 10.1016/j.chembiol.2010.04.012 20534341 PMC7316379

[31] Lyu X, Chastain M, Chai W. Genome-wide Mapping and Profiling of γH2AX Binding Hotspots in Response to Different Replication Stress Inducers. BMC Genomics. 2019;20:579 10.1186/s12864-019-5934-4 31299901 PMC6625122

[32] Kang S, Tsai LT, Zhou Y, Evertts A, Xu S, Griffin MJ, et al Identification of Nuclear Hormone Receptor Pathways Causing Insulin Resistance by Transcriptional and Epigenomic Analysis Nature Cell Biology. 2015;17:44–56. 10.1038/ncb3080 25503565 PMC4281178

[33] Kraushaar DC, Jin W, Maunakea A, Abraham B, Ha M, Zhao K. Genome-wide incorporation dynamics reveal distinct categories of turnover for the histone variant H3.3. Genome Biology. 2013;14(10):R121 10.1186/gb-2013-14-10-r121 24176123 PMC3983652

[34] Rao SSP, Huntley MH, Durand NC, Stamenova EK, Bochkov ID, Robinson JT, et al A 3DMap of the Human Genome at Kilobase Resolution Reveals Principles of Chromatin Looping. Cell. 2014;159(7):1665–80. 10.1016/j.cell.2014.11.021 25497547 PMC5635824

[35] Cai S, Lee CC, Kohwi-Shigematsu T. SATB1 Packages Densely Looped, Transcriptionally Active Chromatin for Coordinated Expression of Cytokine Genes Nature Genetics. 2006;38:1278–88. 10.1038/ng1913 17057718

[36] Christensen J, Cotmore SF, Tattersall P. Minute virus of mice initiator protein NS1 and a host KDWK family transcription factor must form a precise ternary complex with origin DNA for nicking to occur. J Virol. 2001;75(15):7009–17. 10.1128/JVI.75.15.7009-7017.2001 11435581 PMC114429

[37] Christensen J, Tattersall P. Parvovirus initiator protein NS1 and RPA coordinate replication fork progression in a reconstituted DNA replication system. J Virol. 2002;76(13):6518–31. 10.1128/jvi.76.13.6518-6531.2002 12050365 PMC136255

[38] Mincberg M, Gopas J, Tal J. Minute virus of mice (MVMp) infection and NS1 expression induce p53 independent apoptosis in transformed rat fibroblast cells. Virology. 2011;412(1):233–43. 10.1016/j.virol.2010.12.035 21295324

[39] Blackford AN, Jackson SP. ATM, ATR, and DNA-PK: The Trinity at the Heart of the DNA Damage Response. Molecular Cell. 2017;66(6):801–17. 10.1016/j.molcel.2017.05.015 28622525

[40] Melander F, Bekker-Jensen S, Falck J, Bartek J, Mailand N, Lukas J. Phosphorylation of SDT repeats in the MDC1 N terminus triggers retention of NBS1 at the DNA damage-modified chromatin. Journal of Cell Biology. 2008;181(2):213–26. 10.1083/jcb.200708210 18411307 PMC2315670

[41] Nüesch JP, Rommelaere J. A viral adaptor protein modulating casein kinase II activity induces cytopathic effects in permissive cells. Proceedings of the National Academy of Science. 2007;104(30):12482–7. 10.1073/pnas.0705533104 PMC1920537 17636126

[42] Canela A, Maman Y, Jung S, Wong N, Callen E, Day A, et al Genome Organization Drives Chromosome Fragility. Cell. 2017;170(3):507–21. 10.1016/j.cell.2017.06.034 28735753 PMC6133249

[43] Lang F, Li X, Zheng W, Li Z, Lu D, Chen G, et al CTCF prevents genomic instability by promoting homologous recombination-directed DNA double-strand break repair. Proceedings of the National Academy of Science. 2017;114(41):10912–7. 10.1073/pnas.1704076114 PMC5642685 28973861

[44] Minchell NE, Keszthelyi A, Baxter J. Cohesin Causes Replicative DNA Damage by Trapping DNA Topological Stress. Molecular Cell. 2020;78:739–51. 10.1016/j.molcel.2020.03.013 32259483 PMC7242899

[45] Lukas C, Melander F, Stucki M, Falck J, Bekker-Jensen S, Goldberg M, et al Mdc1 couples DNA double-strand break recognition by Nbs1 with its H2AX-dependent chromatin retention. EMBO J. 2004;23(13):2674–83. 10.1038/sj.emboj.7600269 15201865 PMC449779

[46] Spycher C, Miller ES, Townsend K, Pavic L, Morrice NA, Janscak P, et al Constitutive phosphorylation of MDC1 physically links the MRE11-RAD50-NBS1 complex to damaged chromatin. Journal of Cell Biology. 2008;181(2):227–40. 10.1083/jcb.200709008 18411308 PMC2315671

[47] Pomerantz BJ, Mueller CR, Hassell JA. Polyomavirus Large T Antigen Binds Independently to Multiple, Unique Regions on the Viral Genome Journal of Virology. 1983;47:600–10. 10.1128/JVI.47.3.600-610.1983 6312084 PMC255300

[48] Hein J, Boichuk S, Wu J, Cheng Y, Freire R, Jat PS, et al Simian Virus 40 Large T Antigen Disrupts Genome Integrity and Activates a DNA Damage Response via Bub1 Binding. Journal of Virology. 2009;83:117–27. 10.1128/JVI.01515-08 18922873 PMC2612341

[49] Cotsiki M, Lock RL, Cheng Y, Williams GL, Zhao J, Perera D, et al Simian Virus 40 Large T Antigen Targets the Spindle Assembly Checkpoint Protein Bub1 Proceedings of the National Academy of Science. 2004;101:947–52. 10.1073/pnas.0308006100 PMC327122 14732683

[50] Wu X, Avni D,;, Chiba T, Yan F, Zhao Q, Lin Y, et al SV40 T Antigen Interacts With Nbs1 to Disrupt DNA Replication Control Genes and Development. 2004;18:1305–16. 10.1101/gad.1182804 15175262 PMC420356

[51] Jang MK, Shen K, McBride AA. Papillomavirus genomes associate with BRD4 to replicate at fragile sites in the host genome. Plos Pathogens. 2014;10(5):e1004117 10.1371/journal.ppat.1004117 24832099 PMC4022725

[52] Wessel SR, Mohni KN, Luzwick JW, Dungrawala H, Cortez D. Functional Analysis of the Replication Fork Proteome Identifies BET Proteins as PCNA Regulators Cell Reports. 2019;28:3497–509. 31553917 10.1016/j.celrep.2019.08.051 PMC6878991

[53] Schmidt SCS, Jiang S, Zhou H, Willox B, Holthaus AM, Kharchenko PV, et al Epstein–Barr virus nuclear antigen 3A partially coincides with EBNA3C genome-wide and is tethered to DNA through BATF complexes. Proceedings of the National Academy of Science. 2015;112:554–9. 10.1073/pnas.1422580112 PMC4299249 25540416

[54] Cater J, Pintel DJ. The small nonstructural protein of the autonomous parvovirus minute virus of mice (MVM) is required for viral growth in murine cells. Journal of General Virology. 1992;73:1839–43. 10.1099/0022-1317-73-7-1839 1385828

[55] Miller CL, Pintel DJ. The NS2 protein generated by the parvovirus Minute Virus of Mice is degraded by the proteasome in a manner independent of ubiquitin chain elongation or activation. Virology. 2001;285:346–55. 11437668 10.1006/viro.2001.0966

[56] Rasband WS. ImageJ, U. S. National Institutes of Health, Bethesda, Maryland, USA https://imagejnihgov/ij/. 1997–2018.

[57] Langmead B, Salzberg SL. Fast Gapped-Read Alignment With Bowtie 2 Nature Methods. 2012;9:357–9. 10.1038/nmeth.1923 22388286 PMC3322381

[58] Li H, Handsaker B, Wysoker A, Fennell T, Ruan J, Homer N, et al The Sequence Alignment/Map Format and SAMtools. Bioinformatics. 2009;25:2078–9. 10.1093/bioinformatics/btp352 19505943 PMC2723002

[59] Quinlan AR, Hall IM. BEDTools: A Flexible Suite of Utilities for Comparing Genomic Features Bioinformatics. 2010;26:841–2. 10.1093/bioinformatics/btq033 20110278 PMC2832824

[60] Zang C, Schones DE, Zeng C, Cui K, Zhao K, Peng W. A Clustering Approach for Identification of Enriched Domains From Histone Modification ChIP-Seq Data Bioinformatics. 2009;25:1952–8. 10.1093/bioinformatics/btp340 19505939 PMC2732366

[61] Xu S, Grullon S, Ge K, Peng W. Spatial Clustering for Identification of ChIP-enriched Regions (SICER) to Map Regions of Histone Methylation Patterns in Embryonic Stem Cells Methods in Molecular Biology. 2014;1150:97–111. 10.1007/978-1-4939-0512-6_5 24743992 PMC4152844

[62] Stovner EB, Sætrom P. epic2 Efficiently Finds Diffuse Domains in ChIP-seq Data Bioinformatics. 2019;35:4392–3. 30923821 10.1093/bioinformatics/btz232

[63] Afgan E, Baker D, Batut B, van den Beek M, Bouvier D, Čech M, et al The Galaxy platform for accessible, reproducible and collaborative biomedical analyses: 2018 update. Nucleic Acids Research. 2018;46(W1):W537–W44. 10.1093/nar/gky379 29790989 PMC6030816

[64] Bolstad B. preprocessCore: A collection of pre-processing functions. R package version 1500. 2020.

[65] Majumder K, Boftsi M, Pintel DJ. Viral Chromosome Conformation Capture (V3C) Assays for Identifying Trans-interaction Sites between Lytic Viruses and the Cellular Genome. BioProtocols. 2019;9(6):e3198. 10.21769/BioProtoc.3198 PMC6482961 31032382

[66] Naeger LK, Cater J, Pintel DJ. The small nonstructural protein (NS2) of the parvovirus minute virus of mice is required for efficient DNA replication and infectious virus production in a cell-type-specific manner. J Virol. 1990;64(12):6166–75. 10.1128/JVI.64.12.6166-6175.1990 2147041 PMC248791

[67] Ramírez F, Dündar F, Diehl S, Grüning BA, Manke T. deepTools: A Flexible Platform for Exploring Deep-Sequencing Data Nucleic Acids Research. 2014;42:W187–91. 10.1093/nar/gku365 24799436 PMC4086134

