# Supplementary figures and images for: The NS1 protein of the parvovirus MVM Aids in the localization of the viral genome to cellular sites of DNA damage
Source: PLoS Pathog. 2020 Oct 16;16(10):e1009002. doi: 10.1371/journal.ppat.1009002 (PMC7592911; doi:10.1371/journal.ppat.1009002)

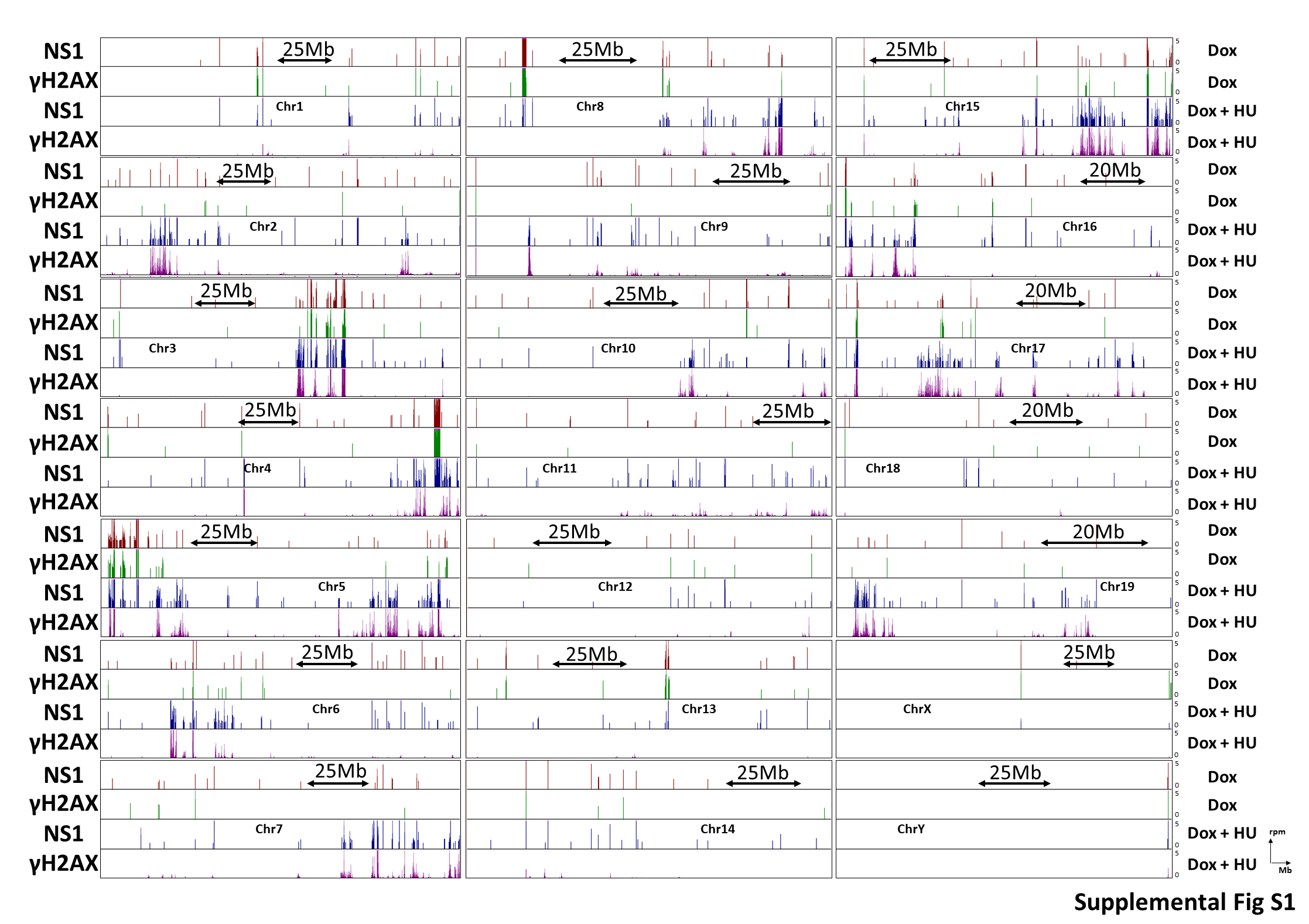

Supplement: S1 Fig — Representative NS1 and γH2AX ChIP-seq data throughout the mouse genome in A9 fibroblasts inducibly expressing NS1 for 24 hours (top 2 panels, in red and green histograms), and in cells pulsed with 1 mM Hydroxyurea for 16 hours starting at 8 hours post-expression (bottom 2 panels, in blue and purple histograms). Y-axis represents quantile normalized reads per million values of ChIP-seq reads for each sample. The chromosome number and scales are indicated for each chromosome throughout the mouse genome. (TIF) [file ppat.1009002.s001.tif]

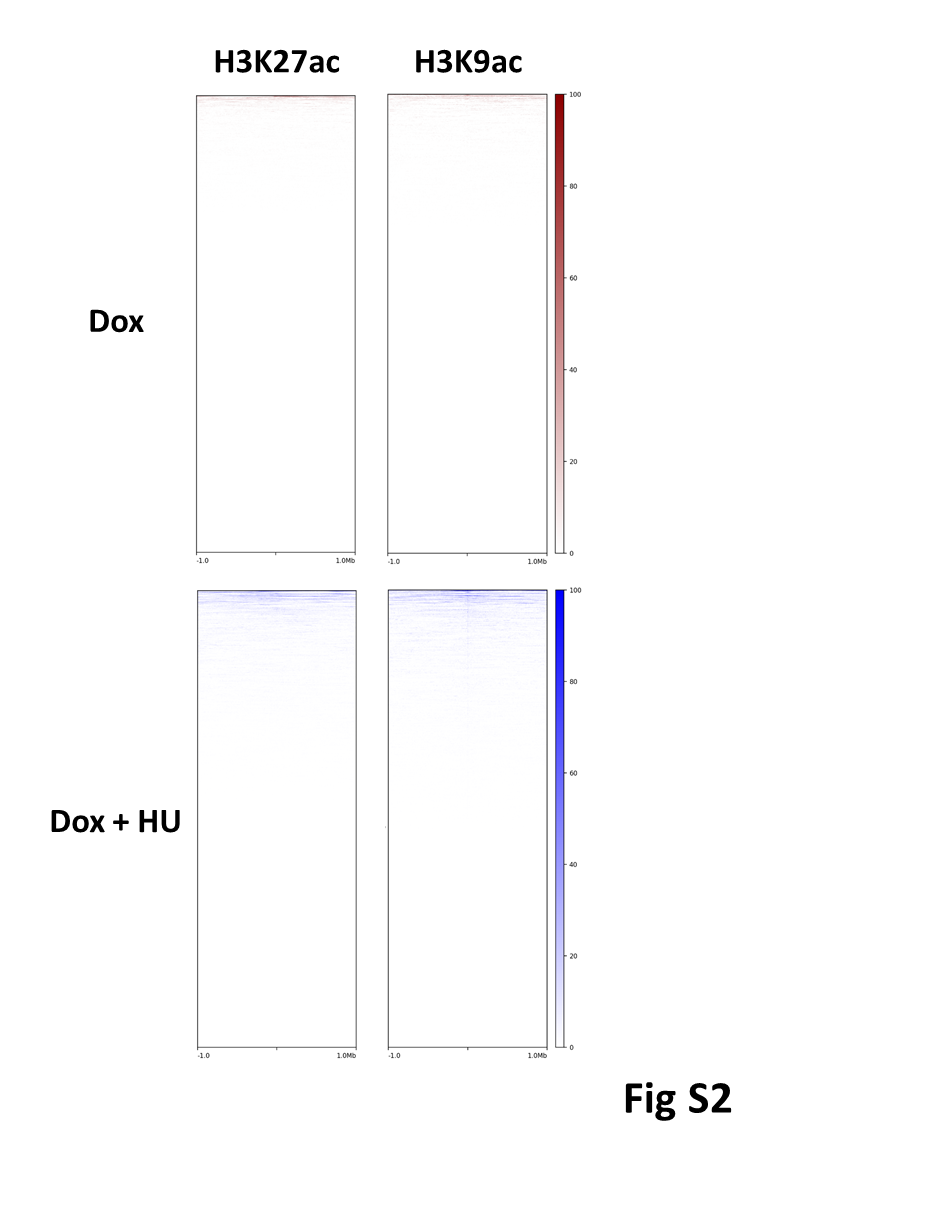

Supplement: S2 Fig — The relative position of NS1 ChIP-seq peaks when NS1 was expressed alone (using doxycycline; top panels in red) and when expressed in the presence of HU (bottom panels in blue) were visualized 1 Mb upstream and downstream of previously published active histone modifications for H3K27ac (left; [32]) and H3K9ac (right; [33]) in 3T3 cells, using deepTools bioinformatics resource on the Galaxy project platform [63,67]. (TIF) [file ppat.1009002.s002.tif]
