# Supplementary material for: The NS1 protein of the parvovirus MVM Aids in the localization of the viral genome to cellular sites of DNA damage
Source: PLoS Pathog. 2020 Oct 16;16(10):e1009002. doi: 10.1371/journal.ppat.1009002 (PMC7592911; doi:10.1371/journal.ppat.1009002)
Supplement: S1 Table — (DOCX) [file ppat.1009002.s003.docx]

| **Primer name** | **Primer sequence (5’ to 3’)** |
| --- | --- |
| *Gene blocks* | |
| MVM 1783-1982; encompassing TGGTTGGT site upstream of MVM p38 with HindIII cloning sites | CATGCTGAGAAGCTTACAGAATGCTTAACATTCATCTAACACATACCTTGCCTGGTGACTTTGGTTTGGTTGACAAAAATGAATGGCCCATGATTTGTGCTTGGTTGGTAAAGAATGGTTACCAATCTACCATGGCAAGCTACTGTGCTAAATGGGGCAAAGTTCCTGATTGGTCAGAAAACTGGGCGGAGCCAAAGGTGCCAACTCCTATAAATAAGCTTCATGCTGAG |
| MVM 1783-1982; with scrambled TGGTTGGT and HindIII cloning sites | CATGCTGAGAAGCTTACAGAATGCTTAACATTCATCTAACACATACCTTGCCTGGTGACTTTGGTTTGGTTGACAAAAATGAATGGCCCATGATTTGTGCGTGTGTGTTAAAGAATGGTTACCAATCTACCATGGCAAGCTACTGTGCTAAATGGGGCAAAGTTCCTGATTGGTCAGAAAACTGGGCGGAGCCAAAGGTGCCAACTCCTATAAATAAGCTTCATGCTGAG |
| *ChIP qPCR primers* | |
| pUC18 upstream of HindIII (F) | TGTAAAACGACGGCCAGTG |
| pUC18 upstream of HindIII (R) | CGGGGATCCTCTAGAGTCG |
| 17qA3.3 (F) | AAGCCAGGGTCATTGACAAC |
| 17qA3.3 (R) | CATGTGTGGCTAGGTCATGG |
| 17qA1 (F) | TAACGACACCGCCATTTACA |
| 17qA1 (R) | TGCCGTAGCTGTTTGCATAG |
| 19qA (F) | AAGTGCCACAGTTGCTCAGA |
| 19qA (R) | CCCACGGCCTTACACATACT |
| 10qD2 (F) | GGCCCAAGTGTGAAGACAGT |
| 10qD2 (R) | CATGCCTTCCTCAAAATGGT |
| 7qF1 (F) | GTGCTTGTGGGTCATCTCCT |
| 7qF1 (R) | TGGAGGTTTTTCTGCTGCTT |
| *ChIP-loop primers* | |
| Taqman probe on pUC18-P38 | TAAATGGGGCAAAGTTCCTG |
| Primer on pUC18-p38 | ACCAATCTACCATGGCAAGC |
| 17qA3.3 HindIII site | TGCCATACGGCTTAATGAGA |
| 17qA1 HindIII site | GGGTAGGGGAGGACTTTCTG |
| *Primers for cloning NS1* | |
| attB1-NS1 (F) | GGGGACAGGTTTGTACAAAAAAGCAGGCTTCACCACCATGGCTGGAAATGCTTACTCTGA |
| attB1R-NS1(R) | GGGGACCACTTTGTACAAGAAAGCTGGGTCACTTGTAGCCAGGAGGCACCCAACCTA |
