# Supplementary material for: The NS1 protein of the parvovirus MVM Aids in the localization of the viral genome to cellular sites of DNA damage
Source: PLoS Pathog. 2020 Oct 16;16(10):e1009002. doi: 10.1371/journal.ppat.1009002 (PMC7592911; doi:10.1371/journal.ppat.1009002)
Supplement: S2 Table — (DOCX) [file ppat.1009002.s004.docx]

**Bioinformatic codes used**

| **Program** | **Function** | **Code** |
| --- | --- | --- |
| Bowtie2 [1] | alignment | bowtie2 -x/storage/htc/biocompute/ircf/dbase/genomes/M_musculus/bowtie2/index/mm10 -U ns1_1.fastq -S ns1_1.sam |
| Samtools [2] | SAM to BAM conversion | samtools view -b -S -o aligned_ns1_1.bam ns1_1.sam  samtools sort -o aligned_sorted_ns1_1.bam aligned_ns1_1.bam |
| BEDtools [3] | BAM to BED conversion | bedtools bamtobed -i aligned_sorted_ns1_1.bam > ns1_1.bed |
| EPIC [4] | Peak calling | epic -t ns1_1.bed -c input.bed -gn mm10 -b BED -o epic_ns1_1 |
| BEDtools [3] | Intersection of BED files | bedtools intersect –a ns1_1.bed –b ns1_2.bed > ns1_1_2.bed |
| BEDtools [3] | Jaccard analysis of overlap extent | bedtools jaccard -a ns1_1_2.bed -b random.bed |

**References**

1. Langmead B, Salzberg SL. Fast Gapped-Read Alignment With Bowtie 2 Nature Methods. 2012;9:357-9. doi: 10.1038/nmeth.1923; PMCID: PMC3322381.

2. Li H, Handsaker B, Wysoker A, Fennell T, Ruan J, Homer N, Marth G, Abecasis G, Durbin R, Subgroup GPDP. The Sequence Alignment/Map Format and SAMtools. Bioinformatics. 2009;25:2078-9. doi: 10.1093/bioinformatics/btp352; PMCID: PMC2723002

3. Quinlan AR, Hall IM. BEDTools: A Flexible Suite of Utilities for Comparing Genomic Features Bioinformatics. 2010;26:841-2. doi: 10.1093/bioinformatics/btq033; PMCID: PMC2832824

4. Stovner EB, Sætrom P. epic2 Efficiently Finds Diffuse Domains in ChIP-seq Data Bioinformatics. 2019;35:4392-3. doi: 10.1093/bioinformatics/btz232.
